# Image Segmentation and Identification of Paired Antibodies in Breast Tissue

**DOI:** 10.1155/2014/647273

**Published:** 2014-07-01

**Authors:** Jimmy C. Azar, Martin Simonsson, Ewert Bengtsson, Anders Hast

**Affiliations:** Centre for Image Analysis, Department of Information Technology, Uppsala University, 75105 Uppsala, Sweden

## Abstract

Comparing staining patterns of paired antibodies designed towards a specific protein but toward different epitopes of the protein provides quality control over the binding and the antibodies' ability to identify the target protein correctly and exclusively. We present a method for automated quantification of immunostaining patterns for antibodies in breast tissue using the Human Protein Atlas database. In such tissue, dark brown dye 3,3′-diaminobenzidine is used as an antibody-specific stain whereas the blue dye hematoxylin is used as a counterstain. The proposed method is based on clustering and relative scaling of features following principal component analysis. Our method is able (1) to accurately segment and identify staining patterns and quantify the amount of staining and (2) to detect paired antibodies by correlating the segmentation results among different cases. Moreover, the method is simple, operating in a low-dimensional feature space, and computationally efficient which makes it suitable for high-throughput processing of tissue microarrays.

## 1. Introduction

The Human Protein Atlas (HPA) project is an initiative that aims to map and explore how known and unknown genes are expressed as proteins at different locations in the human body [1]. This is accomplished by generating antibodies specific for different gene products and staining a multitude of tissues assembled in tissue microarrays. The HPA database is constructed with the use of automated immunostaining machines and slide scanners, which allow for high-throughput protein expression analysis. The database is publicly accessible and provides high resolution images for the staining patterns of different antibodies. Version 12 of the atlas published on December 5, 2013, covers around 81% of all proteins in the human body [2]. The immunohistochemistry has two major steps. First, a primary antibody binds to the protein we wish to detect and then a secondary antibody binds to the primary antibody and produces the stain. The dark brown dye 3,3′-diaminobenzidine (DAB) is commonly used as an antibody-specific stain whereas the blue dye, hematoxylin, is used as a counterstain and is unspecific in its binding. The primary antibody only binds to a small part of the protein, so we can have several different primary antibodies that bind to different parts of the same protein. In this paper, we consider paired primary antibodies, that is, primary antibodies that bind to the same protein but to different sites of the protein. Comparing staining patterns of paired antibodies for a specific protein provides quality control over the binding and the antibodies' ability to identify the target protein correctly and exclusively [3]. Since the antibodies bind to the same protein, they should produce similar staining patterns if they are designed to recognize all isoforms of that protein. However, since they do not bind to the same place, the affinity and specificity of the binding might be different resulting in different intensities in the stain and among different sections. Furthermore, since the antibody only binds to a small part of the protein, a similar part may be present on a different protein, which results in unspecific binding. The Human Protein Atlas includes many tissue types relating to different organs in the human body, and the paired-antibody problem can be studied for any type of tissue and protein. The methods that we develop in this paper are built on general concepts and should be applicable to all the different tissue types. We will however focus on breast tissue in our demonstration of the methods in this paper.

There have been several efforts to segment images of breast tissue sections and quantify the amount of a certain protein; however, these have been overly customized to a specific protein and type of cell segmentation [4–6].

In this paper, we present a method for automated quantification of immunostaining patterns for antibodies in breast tissue. The aim of the method is twofold: (1) to accurately segment and identify staining patterns within breast tissue and quantify the amount of staining and (2) to detect paired antibodies by correlating the segmentation results among different cases. To evaluate the performance of our method we have made use of paired antibodies and consecutive tissue microarray sections which consist of paraffin-embedded tissue samples that are cut into micrometer thin sections before staining. Since the sections are cut one after the other, the same tissue structures and sometimes even parts of the same cell should be present in both sections. The method's basis is unsupervised, relying on clustering. We demonstrate the simplicity of our solution based on an understanding of the problem from a feature space perspective and show the reliability and validity of the segmentation and correlation results. The results are comparable to those of supervised methods that require manual sampling or interactive outlining of sample regions such as in the commercial software ilastik [7] or Genie (Aperio Technologies) [8–10]. However, the difference between our method and the approaches adopted by such software is that our method relies on unsupervised clustering for the segmentation of tissue structures (under light-absorbing stains) as opposed to supervised classification which requires manual interaction and selection of sample regions by a pathologist for the training phase. The only supervised aspect of our method concerns the light-scattering DAB stain and is carried out* a priori* only once, while all subsequent processing is automated. With the software Genie, for example, the interactive process for marking classes is simplified and does not require a detailed outlining of regions of interest; however, the classification is still dependent on a user-defined training set, in addition to continuous user input and feedback. The pathologist needs to train and fine-tune the dataset by adding slides and roughly marking up or modifying regions of interest, as well as often redefining classes. At every iteration, the pathologist must validate the result and accuracy of the algorithm until they are satisfied with the final optimized segmentation. The software is expected to perform very well due to its supervised aspect; however, this is commensurate with the time and effort the pathologist devotes to the process, which may in itself hinder high-throughput processing. Furthermore, another drawback is that Genie's training procedure requires that a complete reoptimization be done with every immunohistochemical stain as well as each tissue type combination [11].

## 2. Materials and Methods

Our dataset consisted of images from the Human Protein Atlas (HPA) database [2].  Table 1 provides a short summary of the dataset we have used in our study, which can also be viewed in more detail in later sections.

**Figure 1:**
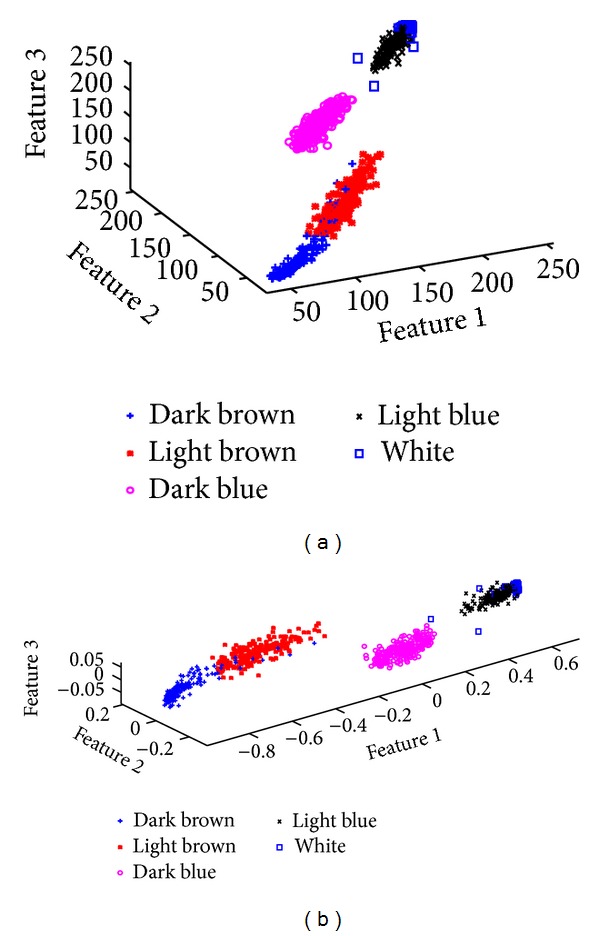
(a) RGB feature space for a balanced dataset; the principal direction of variance extends diagonally. (b) Feature space after PCA; the principal component is aligned to Feature 1, which may now be scaled directly prior to clustering.

Images were rescaled to 25% of their original size using bicubic interpolation. A low pass Gaussian kernel with standard deviation *σ* = 2 was then used to filter the images in order to reduce sensitivity to pixel classification. The filter was applied to each color channel. The number of features in this case is three corresponding to the red, green, and blue channels. Intensity is implicitly included as it represents the distance from the origin to a given pixel location in this 3D feature space (see Figure 1(a)).

**Figure 2:**
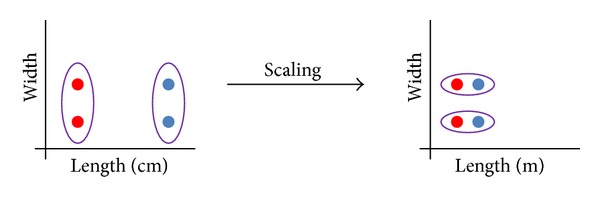
A schematic showing that the effect of scaling a feature with respect to another may influence the clustering result.

The dataset was then normalized by scaling the domain of the features to the range [0,1]. Principal component analysis (PCA) [12] was used to rotate the feature space so that the first feature is aligned with the direction of maximum variance in the data, but no feature reduction was performed (see Figure 1(b)). The resulting feature space in general is sometimes referred to as “stain space” in some literature [13–15]. The purpose of this step was to enable the relative rescaling of features along important directions because this has influence on the clustering algorithm in general as Figure 2 illustrates schematically.

We have also investigated the hue-saturation-value (HSV) color space [16]. The combination of colors present in most regions of the images was in general separable using this color space; however, the class overlap between these regions on one hand and lumen and background regions on the other hand was significant, owing to the fact that these latter regions are prevalently white and therefore composed of various hue components resulting in a significant spread along one dimension, perpendicularly overshadowing other clusters in feature space. A different strategy would have had to be adopted in HSV space. One possibility was to set a threshold or classifier to remove white regions and then treat the remaining clusters separately. However, in the process, stromal regions which are light blue would be compromised, as these lie very close to white in feature space and often contain numerous narrow white regions themselves. This was a main deterrent to using HSV color space.

Prior to designing a method to solve the problem, the scatter plots of sample regions from the data were studied to gain insight, and an interactive function for this purpose was set up. The scatter plots revealed that there are two main branches, one for the brown DAB color and one for the blue hematoxylin color which eventually fades into white (see Figure 1(a)). Along each branch, we may distinguish clusters for dark and light shades of the given color. Note that the separation between the two branches can be achieved with a planar decision surface. We decided to use a supervised method, where a dataset was set up for the brown DAB stain, blue hematoxylin stain, and white background over a set of sample images, and a quadratic classifier [17] in a dissimilarity-based feature space [18] was then trained over the dataset. Once brown DAB regions are identified by the classifier, the remaining data points are clustered using fuzzy *k*-means with *k* = 3 and membership function exponent, *m* = 2. The reason for this strategy comes from the observation that the separation between the two main branches in the scatter plot is relatively large and a supervised classifier, if trained well over a fairly representative training set, would be reliable in classifying new regions since stain variations among images would cause the hematoxylin and DAB branches to shift position, moving slightly closer to each other or away from each other. Employing clustering for identifying simultaneously both the main branches and the clusters along these is unreliable, since this gives the scaling of features large influence over the clustering performance in a way that the scaling would need to change from image to image due to the relative shifting of the two main branches with respect to each other as compared to the shift of clusters along each branch. This makes the latter approach unreliable for automation.

**Figure 3:**
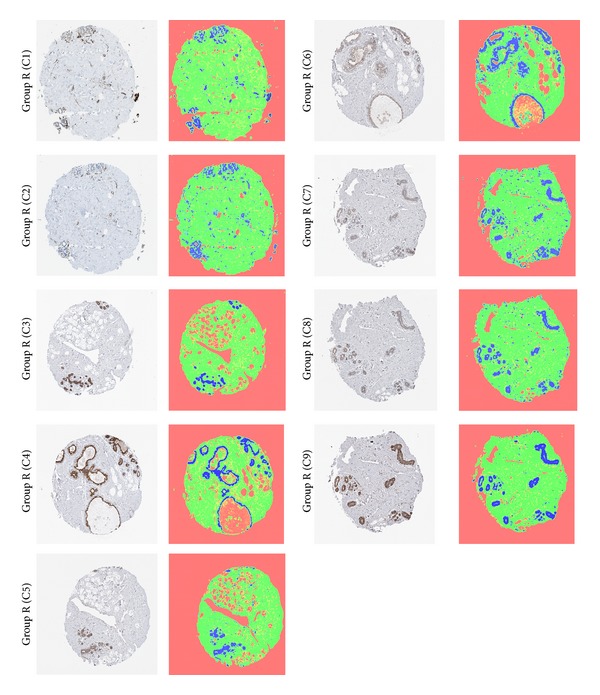
Hematoxylin + DAB staining; sample images and their pseudocolored posterior maps, *k* = 3. The presence of DAB affects the clustering; brown DAB and dark blue regions are grouped into one cluster in the cases above; however, in the general scheme DAB is separated using a trained classifier prior to clustering (see Figure 8). Pseudocolors were assigned as follows: DAB and nuclei appear as blue, stroma appears as green, and lumen/background appears as orange.

Soft labels are extracted following the clustering algorithm in the form of posterior probability maps in the range [0,1], one for each cluster. These are then sorted according to average grayscale intensity to maintain a consistent order when displaying the posterior map images. In an alternative mode of display, a pseudocolored image is generated by multiplying each posterior map with a single-color uniform image and summing up across the maps as shown in Figure 3.

### 2.1. Supervised Classification for Detecting DAB

As mentioned previously, DAB which is a light-scattering stain that does not obey the Beer-Lambert law [19, 20] is identified separately using supervised classification, and DAB-stained regions are thus removed from the image prior to clustering [21]. A training set was constructed for performing pixel classification based on color. This was done through manually selecting regions of interest for each of three classes (blue hematoxylin regions, brown DAB regions, and white lumen regions) across sample images from “Group R” (see Table 1). This resulted in 27000 pixels per class. A quadratic (normal-Bayes) classifier was then trained and tested on this dataset using 10-fold cross-validation. Note that the classifier is only trained once and is not retrained for the other images in the dataset of Table 1. The classifier was not trained in RGB feature space but in a dissimilarity space obtained by a transformation from the original feature space. The dissimilarity space was constructed by first randomly selecting a number of objects from the dataset for each class to form a representation set and then computing the distances between every object in the dataset to each of the prototypes in the representation set. This results in a dissimilarity matrix, and the dimension of the new feature space is the number of objects selected for the representation set. The outcome of training and testing this classifier is discussed in Section 3.

### 2.2. Matching Staining Patterns

One of the purposes of segmenting images corresponding to adjacent breast tissue sections is to determine whether the staining pattern in one image (in terms of class posterior maps) is highly correlated with that of the other image(s). If the correlation is high, the paired antibodies used in the staining are then thought to produce similar staining patterns. On the other hand, suppose there are three paired antibodies A, B, and C that are used each to stain adjacent sections; that is, if antibody A stains a given tissue section, then antibody B stains the next adjacent tissue section and antibody C stains the further next adjacent section. If the correlation measure for the image corresponding to paired antibody A is singularly low with respect to that of B and that of C whereas B and C have high correlation among themselves, antibody A will then be known to produce a different staining pattern from the related B and C antibodies and can therefore be isolated or studied separately.

In order to make a meaningful comparison among two images of tissue sections, the posterior maps have to be registered to provide the best possible alignment since the cutting and physical handling of the sections make it unlikely that they will be perfectly aligned. Instead of using template matching or the sum of absolute differences to register the images, we have used a cross-correlation measure. We address this topic in the following section.

### 2.3. Registration of Posterior Maps

**Figure 4:**
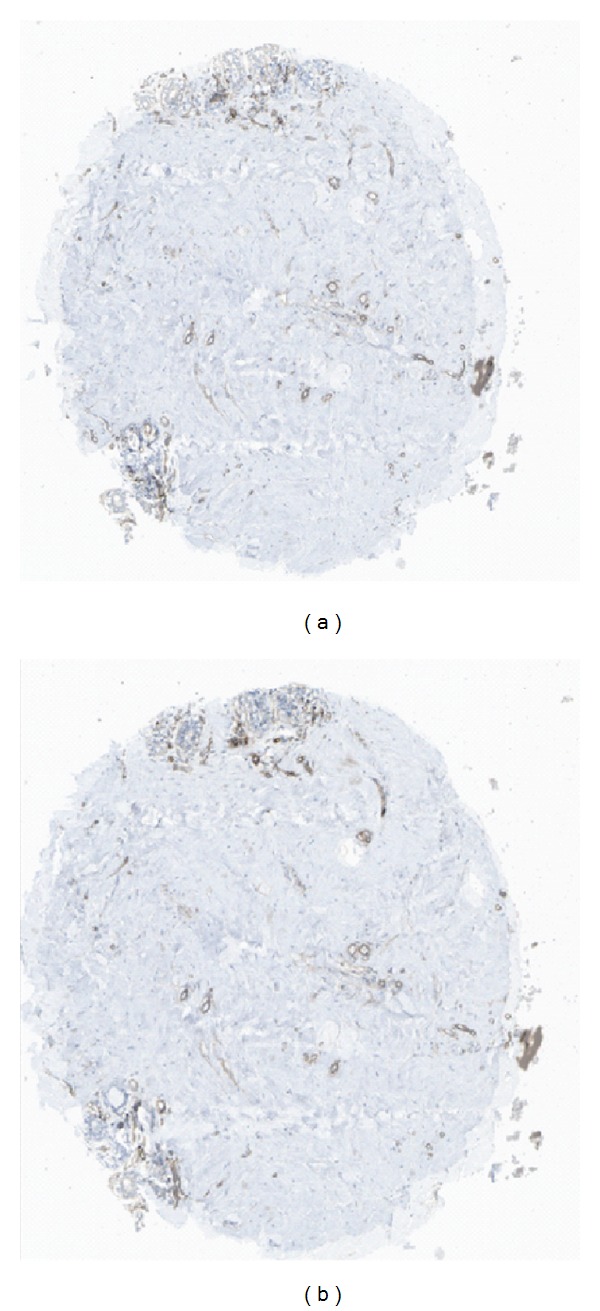
Images of two adjacent sections (cases C1 and C2 of Figure 3) superimposed to show that they are not entirely overlapping but require registration or the use of a cross-correlation measure. (b) The images are registered using normalized cross-correlation.

For the purpose of demonstration, suppose we have decomposed each image into 3 posterior maps. In order to correlate the segmentation of one image with another, we have to do so then using the 3 posterior maps of the first image and the 3 posterior maps of the second, all of which have already been ordered according to average grayscale intensity value. An example of two tissue sections that are not completely aligned is shown in Figure 4(a).

**Figure 5:**
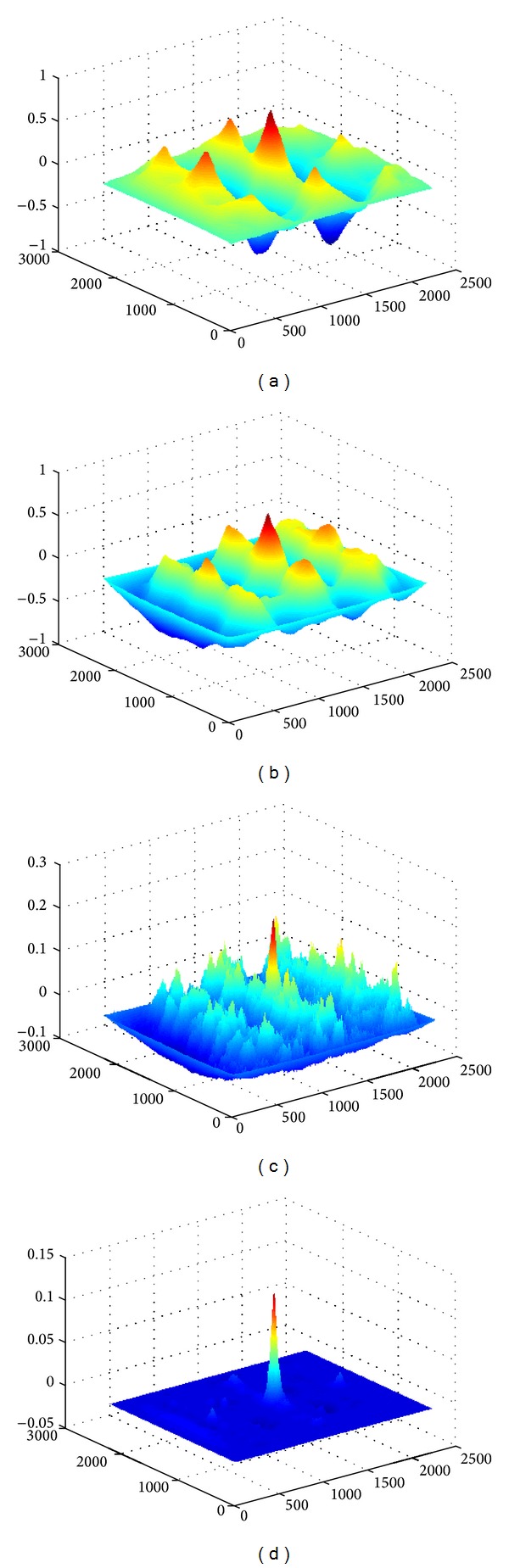
Correlation matrices used for registering the two images in Figure 4. Parts (a), (b), and (c) show surface plots for the correlation coefficient matrices of the posterior maps corresponding to lumen, stroma, and nuclei, respectively. The product of the three matrices is shown in (d). The domain axes represent pixel offset for image rows and columns, whereas the height represents the correlation coefficient value.

In order to register the images, we have used the normalized cross-correlation [22, 23] whereby we correlate each posterior map of one image with the corresponding map of the other image. We thus generate a matrix of correlation coefficients and then consider the absolute value of the coefficients in the matrix and select the highest value (corresponding to the best match). The result consists of three positive coefficients, one for each of the three corresponding pairs of posterior maps. The coefficients are then combined using the product rule into one scalar value in the range [0,1].  Figure 4(b) shows the result of this method for registering images, and Figure 5 shows the correlation matrices for each corresponding pair of posterior maps represented as surface plots for the two case images referred to in Figure 4.

We use the product rule so as to give large influence to individual posterior maps; that is, if one of the posterior maps of the first image does not correlate well with the corresponding map of the second image, then the overall correlation measure will be driven lower by the product. Thus, a high correlation would have to consist of a good match across all the posterior maps or classes. The minimum threshold we have used to define a high correlation value is *T* = (0.5)^*k*=3^ = 0.125; that is, we consider that each pair of corresponding posterior maps should have at least a correlation coefficient of 0.5 resulting in a final value of 0.125 if *k* = 3 as in the cases shown in Table 2. Note that a translation-based registration method such as the one we have used is sufficient for matching the images since orientation differences among adjacent sections are minimal if present, and an expensive rotational correction in this case may not be discernible from a translational one.

**Figure 6:**
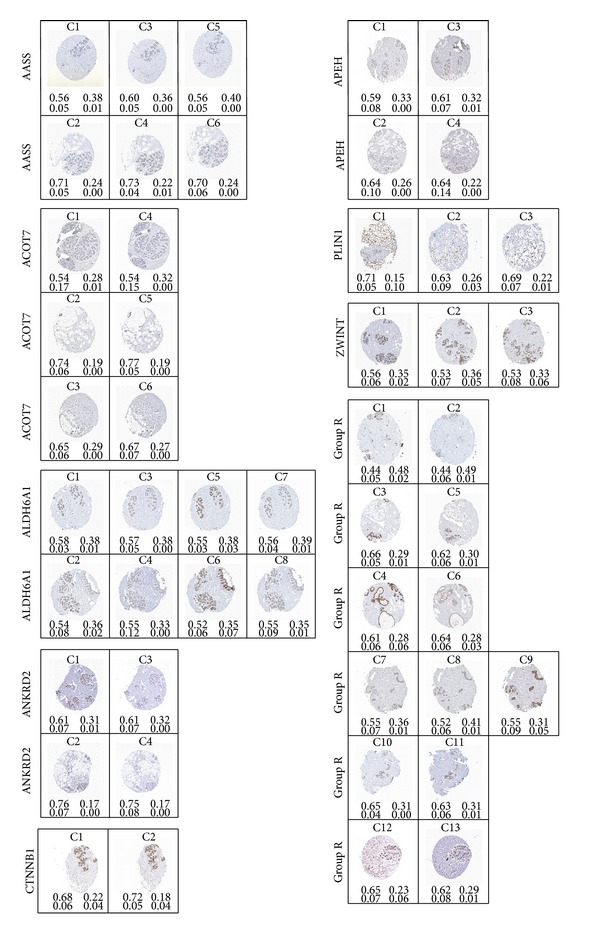
Sample dataset of images of breast tissue microarray sections corresponding to different antigen groups. The numeric values in each case represent the fractional ratio of the four classes in the following order $\left[\begin{array}{cc} \text{Lumen} & \text{Stroma}\\ \text{Nuclei} & \text{DAB} \end{array}\right]$ as quantified by segmentation.

While with *k* = 3 clusters the correlation measure is useful for identifying adjacent sections, in practice such knowledge may be already known; that is to say that the images may have already been grouped or assigned based on adjacent tissue sections during acquisition. The main purpose of performing the matching among images of adjacent tissue sections is then to detect similar and dissimilar staining patterns, in which case DAB becomes an extremely important component when defining these patterns. To summarize the process, after the removal of DAB, the remaining parts of the image are clustered into three classes. Once the cluster labels are obtained, the missing parts (i.e., labels) corresponding to DAB are reintroduced into the image; hence the final segmentation result depicts four classes in total. To this end, four posterior maps were used per image (which includes one for DAB) and the algorithm was tested over 49 images appearing in Figure 6 and has shown accurate and consistent results (see Section 3). Note that making use of all four posterior maps accounts for the hypothetical situation in which DAB may happen to coincidentally spread in a similar manner across two unrelated images that do not correspond to adjacent tissue sections (even though such an occurrence may be rare or unlikely). In addition, our normalized cross-correlation analysis is based on paired-comparisons among images and therefore does not assume a prearrangement of cases or adjacent sections corresponding to the same antibody type. Consequently, we assume nothing is known about the images* a priori*, including whether they belong to adjacent sections or correspond to possible paired antibodies.

In addition to using normalized cross-correlation for matching the staining patterns, the amount of each of the four classes is quantified for each image by applying the maximum* a posteriori* (MAP) rule across the posterior maps to obtain crisp labels and then computing the ratio of the number of pixels of each class label to the total number of pixels in the image. The four classes were named “Lumen,” “Stroma,” “Nuclei,” and “DAB,” the first three being due to the underlying anatomical structures they tend to most often represent in breast tissue images. The numeric values for the four classes sum up to one but were rounded to the second decimal place for convenience. These numeric values appear below each image in Figure 6 in the order specified by the figure legend. Comparing the different cases, we conclude that these values may also be used as a coarse indicator to help identify similar or adjacent sections. Note however that the three assigned classes “Lumen,” “Stroma,” and “Nuclei” refer to regions that have not been positively stained by DAB, and the percentages specified for these in Figure 6 reflect the extent of their presence while excluding any anatomical structures that may have been masked by the DAB stain. The remainder class “DAB” refers to those regions stained by the dye, regardless of what anatomical structures might lie beneath.

## 3. Results and Discussion

### 3.1. Supervised Classification for Detecting DAB

**Figure 7:**
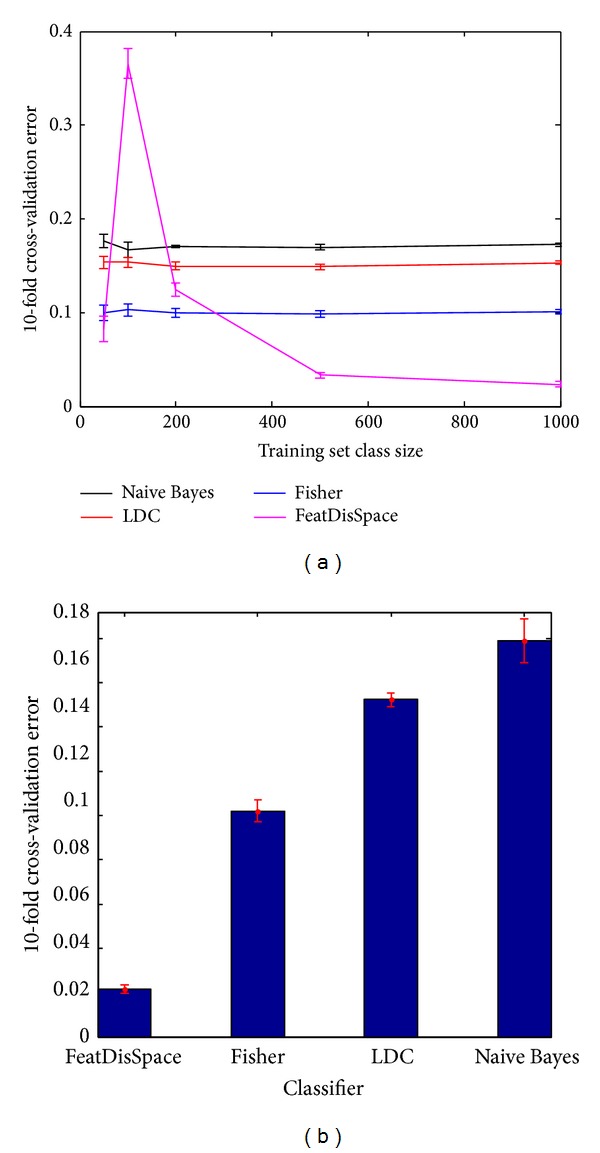
(a) Classifier learning curves. (b) Overall classification error using 10-fold cross-validation.

**Figure 8:**
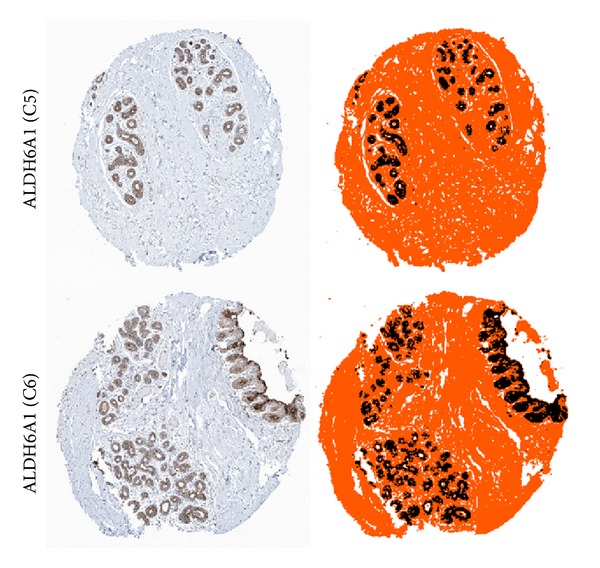
Detection of DAB-stained regions. Pseudocolors were assigned as follows: DAB appears as black, lumen/background appears as white, and the remaining structures appear as orange.

The dissimilarity-based quadratic classifier was trained and tested on the pixel dataset that was introduced previously. This classifier is labeled “FeatDisSpace” in the learning curves of Figure 7(a). The other classifiers are shown as control for comparison, and these are the Naive Bayes classifier, the normal-based linear discriminant classifier (LDC), and the Fisher classifier. The learning curves emphasize the effect of the training set size on the classification error rate. For the chosen classifier, soon after the training set size begins to increase, the error rate drops rapidly. The final error rate reported is that using the entire dataset for 10-fold cross-validation and is shown by the bar graph in Figure 7(b). The results for the detection of DAB-stained regions are shown in Figure 8 for two sample cases.

### 3.2. Unsupervised Classification

In this section we show the effect of clustering into *k* = 3 classes without first removing any DAB regions. The results for sample cases are shown in Figure 3. Clustering with *k* = 4 classes to account for DAB did not prove reliable due to the proximity of the brownish DAB cluster to the other (blue hematoxylin) branch in Figure 1(b) which results in a very sensitive balance required between the scaling of Features 1 and 3. This is one of the reasons why DAB is detected using a trained classifier prior to clustering as shown in Figure 8.

### 3.3. Matching Staining Patterns

In this section we present the results of using the normalized cross-correlation for matching adjacent tissue sections using either three or four posterior maps.

#### 3.3.1. Matching Based on* k* = 3 Posterior Maps (Ignoring DAB)

In summary, the similarity measure between two sets of posterior maps is based on the normalized cross-correlation matrix and the product rule. The method was tested over all the images which have been segmented and their posterior maps were correlated as described previously. Table 2 shows the result of this correlation on the 13 cases of “Group R” and summarizes the findings in a symmetric matrix.

From observing the thirteen sample cases of “Group R” in Figure 6, we note that the following cases form groups of images corresponding to adjacent tissue sections: (C1, C2), (C3, C5), (C4, C6), (C7, C8, C9), (C10, C11), and (C12, C13). That is to say that C1 and C2 represent images of adjacent tissue sections; C3 and C5 likewise represent a pair of adjacent tissue sections, and so forth.

Table 2 shows that indeed the groups (C1, C2), (C4, C6), (C7, C8, C9), (C10, C11), and (C12, C13) have a high similarity measure (>0.125) and therefore represent adjacent sections with similar staining patterns. However, only group (C3, C5) did not have a measure greater than 0.125 and, from Figure 6, we may observe that the reason is due to the missing DAB region in the upper part of image C5 as compared to C3 resulting in a different staining pattern, in addition to some prominent structural differences between these two images.

#### 3.3.2. Matching Based on Four Posterior Maps (Including DAB)

**Figure 9:**
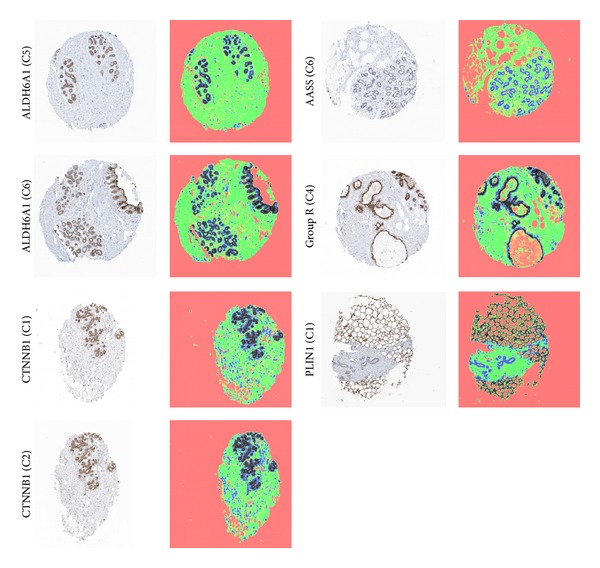
Segmentation of sample cases into four classes: DAB, lumen, stroma, and nuclei. Pseudocolors were assigned as follows: DAB appears as dark-brown, lumen/background appears as orange, stroma appears as green, and nuclei appear as blue.

Sample images along with their segmentation results are shown in Figure 9. The same procedure for matching is performed in this case, however, with four posterior maps for each image instead of three (with the last one corresponding to DAB). The results of these correlation measures for notable cases are shown in Tables 3 and 4. The table section representing group “CTNNB1” shows a high similarity for the two cases C1 and C2, whereas the section representing group “PLIN1” indicates that the three cases C1, C2, and C3 of that group have completely different staining patterns and are unrelated. Similar conclusions may be drawn for the remaining cases.

### 3.4. Quantifying the Proportion of Different Tissue Components and the Proportion Positively Stained

In addition to matching staining patterns and automatically determining whether two sections represent paired antibodies, our method produces quantitative data regarding the relative area covered by each tissue class. As previously mentioned, the fractions of area covered by the classes “Lumen,” “Stroma,” “Nuclei,” and “DAB” are represented by numeric values appearing below each image in Figure 6 and following the order specified by the figure legend.

Even though we have been able to match staining patterns and quantify the amount of each posterior class, we have in doing so considered DAB as one of the main classes without considering the underlying tissue structure hidden beneath the DAB-stained regions. Thus a possible extension of this work is to uncover the nature of the positive, that is, DAB-stained, areas and quantify these in turn as a fraction of the total DAB-stained area in the image or as a fraction of their corresponding structure type. One possible approach is to use, as an additional step, some other types of features such as texture in order to classify the positive areas along with the remaining parts of the image. Another approach would be to analyze the neighborhood of positive areas and attempt to infer the nature of the underlying structures based on the information gathered from these neighborhoods. Yet another possibility is to arrange for a pre-DAB image of the tissue section, that is, prior to DAB staining, in addition to a post-DAB image, that is, following DAB staining. The pre-DAB image may be clustered to identify all tissue structures and assign them a class label, whereas the post-DAB image would provide us with the location of the positive areas, thus directly identifying the underlying structures.

## 4. Conclusions

We have presented a reliable method for segmenting immunostained breast tissue sections and comparing staining patterns for identifying and matching paired antibodies. The algorithm is based on clustering, yet the results are very close and comparable to supervised methods which require manual sampling or interactive delineation of class regions. Moreover, the method works in a low- dimensional space and does not use algorithms with necessarily high time-complexity and is therefore not computationally expensive, requiring on average around 4 seconds per image of size 750 × 750 using Matlab version 7.12 running over an Intel Core i5 CPU with 4 GB RAM and 2.4 GHz clock frequency. This makes the method suitable for high-throughput processing of tissue microarrays. The algorithm was designed based on the exploration and observation of the feature space and was set up using principal component analysis and a modified scaling of the features which allowed the method to be consistent and able to generalize well over cases. One advantage of our method is that, in matching immunostaining patterns in a pair-wise manner, we have used the normalized cross-correlation, not across the original images but rather across the different posterior maps, and combined the values into one similarity measure using the product rule. This allowed us to match staining patterns while taking into account both the DAB staining and the remaining tissue structure areas, in addition to the ability of detecting adjacent sections.

## Figures and Tables

**Table 1:** HPA-based image dataset.

All cell types negative	Gene AASS (6 cases)
	Gene ACOT7 (6 cases)
	Gene ANKRD2 (4 cases)
	Gene APEH (4 cases)

Glandular cells positive; adipocytes negative	Gene ALDH6A1 (8 cases)
	Gene CTNNB1 (2 cases)
	Gene ZWINT (3 cases)

Adipocytes positive; other cell types negative	Gene PLIN1 (3 cases)

Group R (miscellaneous cases C1–C13)	C1, C2: paired antibodies for collagen protein from gene COL15A1
	C3, C5, C4, C6: paired antibodies for protein product from gene FAM54B
	C7, C8, C9: paired antibodies for the cingulin protein from gene CGN
	C10, C11: paired antibodies for the protein product of the gene AC008073.5
	C12, C13: paired antibodies for the protein from gene C16orf70

**Table 2:** Similarity measure between segmented images of “Group R” based on the normalized cross-correlation coefficients among posterior probability maps. The number of classes (maps) is *k* = 3. The matrix is symmetric, and highlighted values are greater than T = (0.5)^3^ indicating high similarity.

	C1	C2	C3	C4	C5	C6	C7	C8	C9	C10	C11	C12	C13
C1	1	**0.1566**	0.0643	0.0490	0.0738	0.0460	0.0694	0.0846	0.0919	0.0584	0.0536	0.0447	0.0602
C2		1	0.0674	0.0605	0.0567	0.0438	0.0719	0.1140	0.1048	0.0518	0.0473	0.0393	0.0447
C3			1	0.0649	0.0925	0.0872	0.0961	0.1057	0.1183	0.0860	0.0690	0.0931	0.0974
C4				1	0.0730	**0.1497**	0.0593	0.0692	0.0669	0.0640	0.0506	0.0517	0.0618
C5					1	0.0717	0.0980	0.0993	0.1156	0.0580	0.0541	0.0563	0.0741
C6						1	0.0701	0.0727	0.0773	0.0644	0.0585	0.0673	0.0831
C7							1	**0.2149**	**0.3417**	0.0450	0.0401	0.0518	0.0594
C8								1	**0.3201**	0.0488	0.0359	0.0564	0.0579
C9									1	0.0495	0.0475	0.0567	0.0660
C10										1	**0.2350**	0.0640	0.0757
C11											1	0.0588	0.0672
C12												1	**0.2805**
C13													1

**Table 3:** Similarity measure between segmented images for sample groups: CTNNB1, PLIN1, and ZWINT.

		C1	C2	C3
CTNNB1	C1	1.0000	**0.0387**	
	C2		1.0000	

PLIN1	C1	1.0000	0.0019	0.0007
	C2		1.0000	0.0026
	C3			1.0000

ZWINT	C1	1.0000	0.0094	0.0107
	C2		1.0000	**0.0364**
	C3			1.0000

The number of posterior maps is 4. The largest significant values are in bold.

**Table 4:** Similarity measure between segmented images for Group R.

	C1	C2	C3	C4	C5	C6	C7	C8	C9	C10	C11	C12	C13
C1	1.0000	**0.0132**	0.0082	0.0037	0.0064	0.0034	0.0049	0.0051	0.0050	0.0038	0.0062	0.0021	0.0068
C2		1.0000	0.0058	0.0035	0.0056	0.0033	0.0048	0.0051	0.0054	0.0048	0.0039	0.0017	0.0045
C3			1.0000	0.0063	**0.0167**	0.0072	0.0058	0.0084	0.0083	0.0062	0.0107	0.0087	0.0114
C4				1.0000	0.0071	**0.0181**	0.0050	0.0068	0.0066	0.0025	0.0044	0.0056	0.0052
C5					1.0000	0.0059	0.0070	0.0074	0.0083	0.0048	0.0071	0.0042	0.0061
C6						1.0000	0.0053	0.0062	0.0071	0.0036	0.0045	0.0055	0.0060
C7							1.0000	**0.0250**	**0.0530**	0.0040	0.0064	0.0036	0.0054
C8								1.0000	**0.0338**	0.0039	0.0056	0.0038	0.0055
C9									1.0000	0.0035	0.0084	0.0046	0.0057
C10										1.0000	**0.0361**	0.0024	0.0073
C11											1.0000	0.0039	0.0075
C12												1.0000	**0.0215**
C13													1.0000

The number of posterior maps is 4. The largest significant values are in bold.

## References

[1] Pontén FK, Schwenk JM, Asplund A, Edqvist P-H (2011). The Human Protein Atlas as a proteomic resource for biomarker discovery. *Journal of Internal Medicine*.

[2] HPA The Human Protein Atlas. http://www.proteinatlas.org/.

[3] Swamidoss IN, Kårsnäs A, Uhlmann V (2013). Automated classification of immunostaining patterns in breast tissue from the human protein atlas. *Journal of Pathology Informatics*.

[4] Tuominen VJ, Ruotoistenmäki S, Viitanen A, Jumppanen M, Isola J (2010). ImmunoRatio: a publicly available web application for quantitative image analysis of estrogen receptor (ER), progesterone receptor (PR), and Ki-67. *Breast Cancer Research*.

[5] Tuominen VJ, Tolonen TT, Isola J (2012). ImmunoMembrane: a publicly available web application for digital image analysis of HER2 immunohistochemistry. *Histopathology*.

[6] Rexhepaj E, Brennan DJ, Holloway P (2008). Novel image analysis approach for quantifying expression of nuclear proteins assessed by immunohistochemistry: application to measurement of oestrogen and progesterone receptor levels in breast cancer. *Breast Cancer Research*.

[7] Sommer C, Straehle C, Kothe U, Hamprecht FA Ilastik: interactive learning and segmentation toolkit.

[8] GENIE, GENetic Imagery Exploitation. http://genie.lanl.gov/.

[9] Perkins SJ, Theiler JP, Brumby SP GENIE: a hybrid genetic algorithm for feature classification in multispectral images.

[10] Harvey NR, Theiler J, Brumby SP (2002). Comparison of GENIE and conventional supervised classifiers for multispectral image feature extraction. *IEEE Transactions on Geoscience and Remote Sensing*.

[11] Rizzardi AE, Johnson AT, Vogel RI (2012). Quantitative comparison of immunohistochemical staining measured by digital image analysis versus pathologist visual scoring. *Diagnostic Pathology*.

[12] Jolliffe IT (2002). *Principal Component Analysis*.

[13] Kriete A, Anderson MK, Love B (2003). Combined histomorphometric and gene-expression profiling applied to toxicology. *Genome Biology*.

[14] Boyce K, Johnson P, Kriete A, Lesniak A, Stone R System and method of generating and storing correlated hyperquantified tissue structure and biomolecular expression datasets.

[15] Stone R, Abdulkarim O, Fuhrman M (2002). Robust stain detection and quantification for histological specimens based on a physical model for stain absorption. *US Patent*.

[16] Agoston MK (2005). *Computer Graphics and Geometric Modeling: Implementation and Algorithms*.

[17] Webb AR (2002). *Statistical Pattern Recognition*.

[18] Duin RP, Pekalska E (2011). The dissimilarity representation for structural pattern recognition. *Progress in Pattern Recognition, Image Analysis, Computer Vision, and Applications*.

[19] Parson WW (2009). *Modern Optical Spectroscopy*.

[20] van der Loos CM (2008). Multiple immunoenzyme staining: methods and visualizations for the observation with spectral imaging. *Journal of Histochemistry and Cytochemistry*.

[21] Gavrilovic M, Azar JC, Lindblad J (2013). Blind color decomposition of histological images. *IEEE Transactions on Medical Imaging*.

[22] Lewis J (1995). Fast template matching. *Vision Interface*.

[23] Haralic RM, Shapiro LG (1992). *Computer and Robot Vision (Volume II)*.

